# Development and validation of a prognostic scoring model for *Mycobacterium avium* complex lung disease: an observational cohort study

**DOI:** 10.1186/s12879-017-2544-0

**Published:** 2017-06-19

**Authors:** Shogo Kumagai, Akihiro Ito, Toru Hashimoto, Satoshi Marumo, Hironobu Tokumasu, Aya Kotani, Haruka Yamaki, Masahiro Shirata, Koji Furuuchi, Motonari Fukui, Tadashi Ishida

**Affiliations:** 1 Department of Respiratory Medicine, Kurashiki Central Hospital, 1-1-1 Miwa, Kurashiki, Okayama, 710-0052 Japan; 2 Respiratory Disease Center, Tazuke Kofukai Medical Research Institute, Kitano Hospital, Osaka, Japan; 3 Department of Consultation, Kurashiki Clinical Research Institute, Okayama, Japan

**Keywords:** Respiratory infections (non-tubeculous), Clinical epidemiology, Clinical respiratory medicine, Bronchiectasis

## Abstract

**Background:**

Patients with *Mycobacterium avium* complex (MAC) lung disease (LD) have a heterogeneous prognosis. This study aimed to develop and validate a prognostic scoring model for these patients using independent risk factors for survival.

**Methods:**

We retrospectively analyzed the data of patients with MAC-LD from two hospitals (cohort 1, *n* = 368; cohort 2, *n* = 118). Cohort 1 was evaluated using a multivariate Cox proportional hazards model to identify independent risk factors for overall survival (OS). A prognostic scoring model composed of these factors was developed, and cohort 1 was stratified into three groups according to risk using the log-rank test. Finally, the prognostic scoring model was validated using the data of cohort 2.

**Results:**

Seven independent risk factors for OS were selected from cohort 1, including the male sex, age ≥ 70 years, the presence of a malignancy, body mass index <18.5 kg/m^2^, lymphocyte count <1000 cells/μL, serum albumin levels <3.5 g/dL, and fibrocavitary disease. The areas under the receiver operating characteristic curves for the prognostic scoring model were 0.84 [95% confidence interval (CI), 0.80 − 0.89] for cohort 1 and 0.84 (95% CI, 0.75 − 0.92) for cohort 2. The 5-year OS rates of patients stratified into low-risk, intermediate-risk, and high-risk groups were 97.6, 76.6, and 30.8%, respectively (*P* < 0.001), in cohort 1, and 97.2, 82.3, and 45.4%, respectively (*P* < 0.001), in cohort 2.

**Conclusions:**

This study is the first to develop and validate a prognostic scoring model for patients with MAC-LD. This model may prove useful in clinical settings and practical in estimating the prognosis.

**Electronic supplementary material:**

The online version of this article (doi:10.1186/s12879-017-2544-0) contains supplementary material, which is available to authorized users.

## Background

Non-tuberculous mycobacteria (NTM) are environmental organisms that cause chronic and progressive lung infection [1]. On the basis of voluntary reporting, the annual incidence of non-tuberculous mycobacterial disease varies from 0.7 − 1.8 cases per 100,000 people worldwide [1]. The *Mycobacterium avium* complex (MAC), including *M. avium* and *M. intracellulare*, is the most common cause of chronic respiratory infections among the NTM species [1–4].

In clinical practice, patients with MAC lung disease (LD) have an extremely heterogeneous prognosis, with some experiencing disease progression to respiratory failure, and others showing stable, indolent disease [2, 5–7]. Although the 2007 American Thoracic Society/Infectious Diseases Society of America (ATS/IDSA) guidelines recommend a three- or four-drug regimen for the treatment of MAC-LD [1], a previous study reported that more than half of patients could be observed without antibiotic treatment [5]. In order to select effective treatment strategies for patients with MAC-LD, it is critical that physicians are able to determine the patient prognosis. Although several factors are reported to be associated with the prognosis of MAC-LD [5], information regarding the prognosis of MAC-LD remains scarce. In addition, there have been no reports of an overall evaluation involving combinations of the prognostic factors of MAC-LD. A prognostic scoring model involving prognostic factors of MAC-LD identified at diagnosis could aid physicians in the prediction of the overall survival (OS) or MAC-specific survival. The purpose of this study was to identify prognostic factors at diagnosis associated with the OS of patients with MAC-LD, and to develop and validate a new prognostic scoring model stratifying the long-term outcomes of MAC-LD.

## Methods

### Patients

This study retrospectively reviewed patients aged >18 years who were newly diagnosed with MAC-LD according to the 2007 ATS/IDSA diagnostic criteria [1] between January 2006 and December 2011 at Kurashiki Central Hospital (cohort 1) and Kitano Hospital (cohort 2). We excluded patients who had already received treatment of MAC-LD at other institutions, or who were HIV-infected.

### Study design

This study was a retrospective observational cohort study. The study protocol was approved by the ethical committees of Kurashiki Central Hospital and Kitano Hospital, and was performed in accordance with the Declaration of Helsinki (IRB number: 2091). Due to the retrospective nature of this study, written informed consent was waived. Referring to previous reports [5, 6], the following clinical characteristics were reviewed from the available clinical records: sex, age, smoking history, systemic comorbidities [chronic heart diseases, diabetes mellitus (DM), collagen vascular diseases, malignancy, chronic liver diseases, neurological diseases, and chronic renal diseases], respiratory comorbidities (old pulmonary tuberculosis, emphysema, interstitial pneumonia, lung cancer, asthma, and bronchiectasis), computed tomography findings [nodular bronchiectatic (NB) disease, fibrocavitary (FC) disease, NB/FC disease, and other diseases], body mass index (BMI), body temperature, lymphocyte counts, hemoglobin (Hb), serum albumin (Alb), and C-reactive protein (CRP), sensitivity of MAC to macrolide.

The OS was measured from the date of diagnosis of MAC-LD until the date of death from any cause, or censored on the date on which the patient was last known to be alive. The MAC-specific survival was measured from the date of diagnosis of MAC-LD until the date of death from MAC-LD, or censored on the date on which the patient was last known to be alive or died from other causes than MAC-LD.

### Statistical analysis

Categorical variables are presented as frequency (percentage), and continuous variables are shown as the mean ± standard deviation. OS rates were estimated using the Kaplan-Meier analysis [8]. Differences between survival curves were evaluated for statistical significance using the two-tailed log-rank test. We used the method of Holm to account for multiple testing [9]. Univariate and multivariate prognostic analyses were performed to identify independent risk factors associated with OS using the Cox proportional hazards model. Risk factors are chosen, referring to a previous report [5]. We added the details of comorbidities to the risk factors of the previous report. In the multivariate analysis, a stepwise backward procedure was employed to derive a final model of the variables that had a significant independent association with OS. To remove a variable from the model, the corresponding *P*-value had to be >0.05. The patients were classified into three risk groups (high, intermediate, or low) according to a prognostic scoring model composed of independent prognostic factors identified in the multivariate analysis. Patients whose 5-year mortality rate are less than and equal to 60.0%, more than 60% and less than and equal to 90%, or more than 90% and less than or equal to 100% are classified into the high-risk group, intermediate-risk group, low-risk group, respectively. Receiver operating characteristic (ROC) curve analysis and the area under the ROC curve (AUC) were used to evaluate the ability of the prognostic scoring model to predict all-cause mortality. In comparison of the AUCs for two ROC curves, DeLong’s test was employed. Statistical analyses were performed using the statistical software R version 2.13.1 (R Foundation for statistical computing, Vienna, Austria). All *P*-values are 2-sided, and *P* < 0.05 was considered statistically significant.

## Results

### Patient characteristics

A total of 486 patients were included in this study. The baseline characteristics of patients included in this study are shown in Table 1. The median follow-up duration was 42 months (0.1-112 months) for cohort 1, and 71 months (0.2-120 months) for cohort 2. The 5-year OS rates for cohorts 1 and 2 were 77.5 and 85.3%, respectively. Macrolide resistant diseases were not seen in this study.

**Table 1:** Patient characteristics

	Cohort 1, *N* = 368	Cohort 2, *N* = 118	*P-*value
Male	151 (41.0)	45 (38.1)	0.592
Age, years	72 ± 10	70 ± 10	0.019
Smoking history			0.264
Current	20 (5.4)	4 (3.4)	
Past	224 (60.9)	76 (64.4)	
Never	114 (31.0)	38 (32.2)	
Unknown	10 (2.7)	0 (0.0)	
Comorbidity			
Respiratory diseases	110 (29.9)	41 (34.7)	0.361
Old pulmonary tuberculosis	31 (8.4)	24 (20.3)	0.001
Emphysema	40 (10.9)	14 (11.9)	0.739
Interstitial pneumonia	29 (7.9)	6 (5.1)	0.413
Lung cancer	31 (8.4)	6 (5.1)	0.318
Asthma	12 (3.3)	6 (5.1)	0.402
Bronchiectasis	20 (5.4)	5 (4.2)	0.811
Systemic diseases			
Chronic heart diseases	45 (12.2)	18 (15.3)	0.431
Diabetes mellitus	40 (10.9)	10 (8.5)	0.492
Collagen vascular diseases	27 (7.3)	13 (11.0)	0.247
Malignancy^a^	86 (23.4)	27 (22.9)	0.999
Chronic liver diseases	8 (2.2)	6 (5.1)	0.115
Neurological diseases	33 (9.0)	12 (10.2)	0.716
Chronic renal diseases	7 (1.9)	9 (7.6)	0.005
Steroid use	14 (3.8)	7 (6.0)	0.306
Immunosuppressant use^b^	19 (5.2)	5 (4.3)	0.811
BMI, kg/m^2^	19.6 ± 3.4	20.3 ± 3.2	0.090
BT, °C	36.7 ± 0.6	36.7 ± 0.9	0.735
Laboratory findings			
Lymphocytes,/μL	1400 ± 680	1300 ± 480	0.014
Hb, g/dL	12.4 ± 1.8	13 ± 1.6	0.001
Alb, g/dL	3.9 ± 0.6	4.2 ± 0.4	<0.001
CRP, mg/dL	1.7 ± 3.5	3.7 ± 7.4	<0.001
Cre, mg/dL	0.79 ± 0.73	0.9 ± 0.99	0.206
Diagnosis methods			<0.001
Sputum	263 (71.5)	115 (97.5)	
Bronchoscopy	105 (28.5)	3 (2.5)	
Bacteriological examinations			
Smear positive	64 (17.4)	38 (32.2)	0.001
Culture ≥2+	74 (20.1)	NE	
Macrolide resistant	0 (0.0)	0 (0.0)	NE
Radiological findings			0.337
NB	298 (81.0)	101 (85.6)	
FC	41 (11.1)	14 (11.9)	
FC/NB	6 (1.6)	0 (0.0)	
Other^c^	35 (9.5)	3 (2.5)	
Deaths	75 (20.5)	20 (16.9)	0.505
Causes of death			0.864
MAC-specific death	29 (38.7)	9 (45.0)	
Malignancy	20 (26.7)	4 (20.0)	
Other	20 (26.7)	5 (25.0)	
Unknown	6 (0.1)	1 (5.0)	

Data are n (%) or mean ± standard deviation

*BMI* body mass index, *BT* body temperature, *Hb* hemoglobin, *Alb* serum albumin, *CRP* C-reactive protein, *Cre* creatinine, *NE* not evaluated, *NB* nodular/bronchiectatic disease, *FC* fibrocavitary disease

^a^Malignancy includes lung cancer

^b^Immunosuppressants included methotrexate, cyclosporin, azathioprine, cyclophosphamide, tacrolimus, etanercept, salazopyrin, mizoribine, and bucillamine

^c^Other included unclassifiable and disseminated diseases

### First-line treatment

First-line treatment regimens including more than one regimen are shown in Table 2. A total of 235 patients (48.4%) received first-line treatments. The most frequently prescribed treatment regimen in both cohorts was the combination of clarithromycin (CAM), ethambutol (EB), and rifampicin (RFP) (cohort 1, 79.3%; cohort 2, 59.1%).

**Table 2:** First-line treatment regimens

	Cohort 1	Cohort 2
Patients who received treatments	169 (100.0)	66 (100.0)
CAM + EB + RFP	134 (79.3)	39 (59.1)
CAM + RFP	30 (17.8)	6 (9.1)
CAM + EB + RFP + SM	2 (1.2)	2 (3.0)
CAM + EB	1 (0.6)	6 (9.1)
CAM + RFP + NQ	1 (0.6)	5 (7.6)
CAM + EB + NQ	0 (0.0)	2 (3.0)
CAM + EB + RFP + NQ	0 (0.0)	2 (3.0)
Other regimens	1(0.6)	4 (6.1)

Data are n (%)

*CAM* clarithromycin, *RFP* rifampicin, *EB* ethambutol, *SM* streptomycin, *NQ* new quinolones

### Prognostic analyses for OS

A univariate analysis identified 14 significant risk factors associated with OS in cohort 1: the male sex, age ≥ 70 years, ever smokers, respiratory diseases, DM, malignancies, neurological diseases, chronic renal diseases, BMI <18.5 kg/m^2^, lymphocyte count <1000 cells/μL, Hb <10.0 g/dL, Alb <3.5 g/dL, CRP ≥1.0 mg/dL, and FC disease (Table 3). The multivariate analysis identified seven significant negative prognostic factors for OS, including the male sex, age ≥ 70 years, the presence of a malignancy, BMI <18.5 kg/m^2^, lymphocyte count <1000 cells/μL, Alb <3.5 g/dL, and FC disease.

**Table 3:** Prognostic analyses of risk factors for overall survival

	Univariate analysis			Multivariate analysis		
Variables	HR	95% CI	*P*-value	HR	95% CI	*P*-value
Male	3.98	2.44 − 6.45	<0.001	3.16	1.88 − 5.31	<0.001
Age ≥ 70 years	3.54	1.98 − 6.33	<0.001	2.15	1.18 − 3.93	0.012
Ever-smokers	3.41	2.12 − 5.46	<0.001			
Respiratory diseases	3.76	2.38 − 5.95	<0.001			
Diabetes mellitus	2.50	1.45 − 4.30	<0.001			
Malignancy^a^	3.25	2.05 − 5.14	<0.001	1.98	1.23 − 3.18	0.005
Neurological diseases	2.13	1.15 − 4.30	<0.001			
Chronic renal diseases	2.92	0.92 − 9.29	0.07			
BMI <18.5 kg/m^2^	2.34	1.48 − 3.71	<0.001	2.12	1.29 − 3.48	0.003
Lymphocytes <1000/μL	4.30	2.73 − 6.77	<0.001	2.36	1.47 − 3.78	<0.001
Hb <10.0 g/dL	2.30	1.21 − 4.36	0.011			
Alb <3.5 g/dL	6.66	4.19 − 10.6	<0.001	3.93	2.42 − 6.40	<0.001
CRP ≥1.0 mg/dL	4.28	2.71 − 6.76	<0.001			
FC pattern	2.87	1.65 − 5.00	<0.001	1.96	1.10 − 3.52	0.024

*95% CI* 95% confidence interval, *HR* hazard ratio, *BMI* body mass index, *Hb* hemoglobin, *Alb* albumin, *CRP* C-reactive protein, *FC* fibrocavitary disease

^a^Malignancy included lung cancer

### Prognostic scoring model composed of significant negative prognostic factors

In developing a prognostic scoring model, we examined two prognostic scoring models consisting of the seven independent factors identified in the multivariate analysis (Table 4). In the prognostic scoring model 1, we allocated one point for all the seven factors, while in the prognostic scoring model 2 we allocated three points for male sex, four points for hypoalbuminemia, and two points for the other five factors, according to hazard ratios (HRs) shown in the multivariate analysis. The comparisons of the AUCs for the ROC curves of both prognostic scoring models revealed no significant differences between the two prognostic models (cohort 1; 0.84 vs. 0.85; *P* = 0.310). So, considering clinical utility and ease for calculation, we adopted the prognostic scoring model 1. We stratified patients according to the following three risk groups: low-risk (0–1 point), intermediate-risk (2–3 points), and high-risk (≥4 points).

**Table 4:** Development of a prognostic scoring model

Variables	Prognostic scoring model 1	Prognostic scoring model 2
Male	1 point	3 points
Age ≥ 70 years	1 point	2 points
Malignancy^a^	1 point	2 points
BMI <18.5 kg/m^2^	1 point	2 points
Lymphocytes <1000/μL	1 point	2 points
Alb <3.5 g/dL	1 point	4 points
FC pattern	1 point	2 points
	Total scores	
Risk groups	Prognostic scoring model 1	
Low-risk	0 − 1 point	
Intermediate-risk	2 − 3 points	
High-risk	≥4 points	

*95% CI* 95% confidence interval, *HR* hazard ratio, *BMI* body mass index, *Alb* albumin, *FC* fibrocavitary disease

^a^Malignancy included lung cancer

**Fig. 1:**
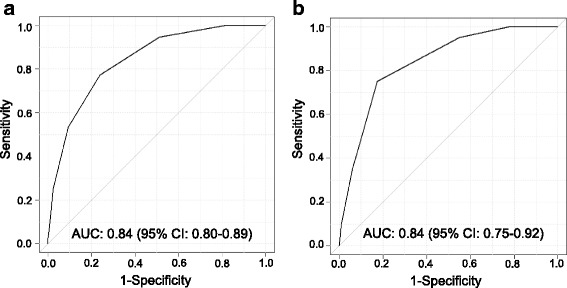
Receiver operating characteristic curves for the prognostic scoring model predicting all-cause mortality for (**a**) cohort 1 and (**b**) cohort 2. AUC, area under the curve; 95% CI, 95% confidence interval

We constructed ROC curves to assess the ability of the prognostic scoring model to predict all-cause mortality in cohort 1 (Fig. 1a) and cohort 2 (Fig. 1b). The AUCs for the ROC curves were 0.84 [95% confidence interval (CI), 0.80 − 0.89] for cohort 1 and 0.84 (95% CI, 0.75 − 0.92) for cohort 2. Survival according to the prognostic scores is shown in Additional file 1: Table S1. A higher prognostic score tended to be associated with a worse prognosis.

### Analyses of the outcomes of the low-risk, intermediate-risk, and high-risk groups according to the prognostic scoring model

**Fig. 2:**
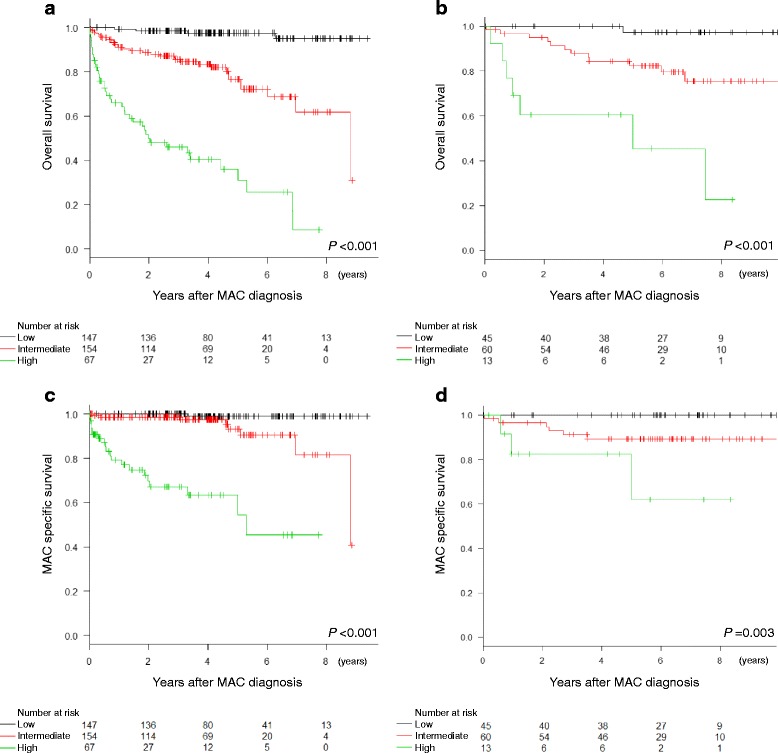
Kaplan-Meier analyses of overall survival and MAC-specific survival in (**a**, **c**) cohort 1 and (**b**, **d**) cohort 2 patients stratified into low-risk, intermediate-risk, and high-risk groups according to the prognostic scoring model. MAC, *Mycobacterium avium* complex

The Kaplan-Meier analysis of the OS of patients in cohort 1 demonstrated significant differences in the outcomes among the three risk groups (*P* < 0.001 for all comparisons; Fig. 2a). The 5-year OS rates were 97.6% (147 patients), 76.6% (154 patients), and 30.8% (67 patients) for the low-risk, intermediate-risk, and high-risk groups, respectively. In cohort 2, the Kaplan-Meier analysis showed significant differences in the OS among the three groups (*P* < 0.001; intermediate-risk vs. low-risk, *P* = 0.007; high-risk vs. low-risk, *P* < 0.001; high-risk vs. intermediate-risk, *P* = 0.002; Fig. 2b). The 5-year OS rates were 97.2% (45 patients), 82.3% (60 patients), and 45.4% (13 patients) for the low-risk, intermediate-risk, and high-risk groups, respectively. The Kaplan-Meier analysis of the MAC-specific survival of patients in cohort 1 demonstrated significant differences in the outcomes among the three risk groups (*P* < 0.001; intermediate-risk vs. low-risk, *P* = 0.004; high-risk vs. low-risk, *P* < 0.001; high-risk vs. intermediate-risk, *P* < 0.001; Fig. 2c). The 5-year MAC-specific survival rates were 99.0, 93.3, and 54.4% for the low-risk, intermediate-risk, and high-risk groups, respectively. In cohort 2, the Kaplan-Meier analysis showed significant differences in the MAC-specific survival among the three groups (*P* = 0.003; intermediate-risk vs. low-risk, NS; high-risk vs. low-risk, *P* < 0.001; high-risk vs. intermediate-risk, NS; Fig. 2d). The 5-year MAC-specific survival rates were 100.0, 89.4, and 61.9% for the low-risk, intermediate-risk, and high-risk groups, respectively.

In the analysis of OS, HRs of the intermediate-risk or high-risk groups compared with the low-risk group were evaluated in cox regression analyses. In cohort 1, the HR of the intermediate-risk group vs. the low-risk group was 9.65 (95% CI, 3.40 − 27.4; *P* < 0.001), and that of the high-risk group vs. the low-risk group was 46.0 (95% CI, 16.3 − 130.3; *P* < 0.001). In cohort 2, the HR of the intermediate-risk group vs. the low-risk group was 9.83 (95% CI, 1.28 − 75.7; *P* = 0.028), and that of the high-risk group vs. the low-risk group was 42.2 (95% CI, 5.16 − 345.4; *P* < 0.001).

### Treatment and outcomes

**Fig. 3:**
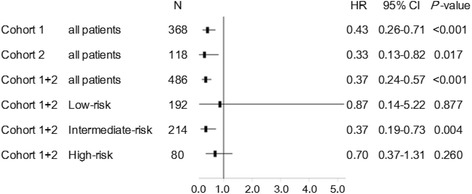
Impacts of treatment with more than one regimen on OS in each subgroup were shown in the forest plot

Forest plots showed the HRs of OS for patients with any treatment compared to those without treatment in subgroups of the present study (Fig. 3). The HR of the patients with any treatment compared to those without treatment in the whole cohort (cohorts 1 and 2) was 0.37 (95% CI: 0.24–0.57; *P* < 0.001). Of the three risk groups, the HR in the intermediate-risk group was the lowest (0.37, 95% CI; 0.19–0.73, *P* = 0.004).

## Discussion

The MAC has emerged as an increasingly prevalent pathogen in respiratory infections [3]. The long-term outcomes of patients with infections caused by MAC vary from chronic indolence to rapid progression [2, 5–7, 10]. Therefore, a prognostic scoring model is required to predict the heterogeneous prognosis of MAC-LD at diagnosis in clinical practice. In order for the prognostic scoring model to be clinically useful and easy to calculate, we investigated prognostic factors of OS which are easily available in the clinical setting for inclusion in the prognostic scoring model. Seven independent prognostic factors were identified in the multivariate analysis, including the male sex, advanced age, malignancy, low BMI, low lymphocyte counts, hypoalbuminemia, and FC disease. These factors were used to develop the prognostic scoring model that significantly stratified the OS of patients with MAC-LD into three groups according to risk (high/intermediate/low), the results of which were validated using an independent cohort. Further prospective studies are required to assess the long-term effectiveness of the treatment of MAC-LD. To our knowledge, this is the first study to devise a prognostic scoring model stratifying not only OS but also MAC-specific surival of patients with MAC-LD. This is very important in helping patients and physicians to select an optimal management strategy.

Of the systemic comorbidities, a malignancy was the most prevalent in this study; patients with lung cancer constituted 7.6% of the study population. Lande et al. conducted a retrospective analysis of patients with MAC-positive respiratory cultures and newly diagnosed lung cancer [11]. They concluded that the presence of MAC in the respiratory cultures of patients with lung cancer was particularly associated with squamous cell carcinomas located in the periphery of the lung. Chronic lung infections caused by mycobacterial organisms can stimulate proinflammatory reactions that cause extensive damage to the surrounding lung and bronchiolar tissues [12–15]. It has been reported that chronic inflammatory processes nurture the development of malignancies in tissues before evident tumors are established [16], and may make tumor progression possible by promoting immune tolerance [17]. Therefore, when considering the treatment strategies for MAC-LD, physicians should be aware of the association between MAC and malignancy.

The 5-year survival rate of patients with MAC-LD in the present study was 77.5% in cohort 1 and 85.3% in cohort 2. These results were consistent with previous reports, which reported 5-year mortality rates ranging from 23.9 to 39.7% [2, 5, 18, 19]. There are few reports assessing the prognostic factors of MAC-LD. Hayashi et al. reported that the male sex, age ≥ 70 years, the presence of systemic and/or respiratory comorbidities, the radiographic features of FC, FC + NB, or other diseases, BMI <18.5 kg/m^2^, Hb <10 g/dL, Alb <3.5 g/dL, and an erythrocyte sedimentation rate (ESR) ≥50 mm/h were negative prognostic factors for all-cause mortality in a multivariate analysis [5]. Ito et al. evaluated the data of 78 patients with definite MAC disease, and identified two independent factors for 5-year all-cause mortality: a high Charlson comorbidity index and cavity lesions [19]. In the present study, only FC disease, and not FC/NB disease, was included in the multivariate analysis, because FC disease showed the worst prognosis in the radiographic patterns (data not shown). Furthermore, ESRs were excluded from the analysis of prognosis because of the small number of patients who were evaluated for ESR at diagnosis. The multivariate analysis identified that a lymphocyte count <1000 cells/μL and the presence of a malignancy were independent prognostic factors for the OS, in addition to the male sex, age ≥ 70 years, radiographic features of FC diseases, a BMI <18.5 kg/m^2^, and an Alb <3.5 g/dL. Lymphocyte count would be assumed to reflect host immunity. Malignancy is in itself a progressive and life-threatening disease. Besides, malignancy and its treatment often cause immunosuppression [20, 21], which might worsen various infections.

The most prescribed regimen in this study was the 2007 ATS/IDSA guidelines recommended regimen (CAM + RFP + EB). The 2007 ATS/IDSA guidelines suggests that the choice of therapeutic regimen for a specific patient depends to some degree on the goals of therapy for that patient, and that especially in treating older frail patients with comorbid conditions who have difficulty tolerating multidrug MAC treatment regimens, less aggressive or even suppressive treatment strategies should be considered [1]. The effect of multidrug treatment on long-term outcome may be difficult to evaluate, because treatment was introduced according to the decision of each physician, and because treatment regimens and durations were not uniform across patients. In this study, however, the patients with treatment experienced favorable OS as compared to those without treatment. In particular, subgroup analyses showed that of the three risk groups, patients in the intermediate-risk group received the best benefits of treatment. In the low-risk group, HR of patients who received treatment is the highest of all the subgroups (*P* = 0.877). Perhaps, the low-risk group patients could be observed with very good prognosis. The high-risk group patients might be so frail and likely to die of other causes including malignancy rather than MAC-LD. This result suggests that physicians should not miss the chance to treat patients especially in the intermediate-risk group and that the low-risk group patients could be observed without treatment. However, it should be kept in mind that continuous assessment is required even in low-risk group because individual patients may have more rapid clinical deterioration than others (and perhaps fall into a higher risk group). This prognostic scoring model should be used, auxiliary to the ATS/IDSA guidelines. An individual risk-benefit assessment of treatment of MAC-LD is necessary for all the patients regardless of risk groups.

One limitation of this study was that this was a retrospective study. Further prospective studies are required to assess the long-term effectiveness of the treatment of MAC-LD. Differences in the background patient characteristics or treatment strategies of MAC-LD among institutions would exist. Therefore, the results of this study should be validated in other multi-center studies. In addition, as patients with suspected MAC-LD who were unable to expectorate sputum were examined by bronchoscopy, the date of diagnosis might have been earlier for these patients and, thus, the 5-year survival rate might have been better.

## Conclusions

This study was the first to develop and validate a prognostic scoring model for MAC-LD, which consisted of seven independent prognostic factors identified in a multivariate analysis. These factors included the male sex, age ≥ 70 years, the presence of a malignancy, BMI <18.5 kg/m^2^, lymphocyte counts <1000 cells/μL, Alb <3.5 g/dL, and FC disease. This is an easy-to-calculate, clinically-relevant prognostic scoring model, which may help physicians to determine the prognosis of patients with MAC-LD and thereby might guide the selection of optimal treatment strategies.

## Additional file

Additional file 1: Table S1. Survivals according to scores of Mycobacterium avium complex lung disease prognostic index. Survivals (1-year, 3-year, and 5-year) according to the prognostic scores are shown. (DOCX 14 kb)

## Abbreviations

Alb: Albumin
ATS/IDSA: American Thoracic Society/Infectious Diseases Society of America
AUC: Area under the ROC curve
BMI: Body mass index
CAM: Clarithromycin
CI: Confidence interval
CRP: C-reactive protein
DM: Diabetes mellitus
EB: Ethambutol
FC: Fibrocavitary
Hb: Hemoglobin
HRs: Hazard ratios
LD: Lung disease
MAC: *Mycobacterium avium* complex
NB: Nodular bronchiectatic
NTM: Non-tuberculous mycobacteria
OS: Overall survival
RFP: Rifampicin
ROC: Receiver operating characteristic
