# Supplementary material for: Development and validation of a prognostic scoring model for Mycobacterium avium complex lung disease: an observational cohort study
Source: BMC Infect Dis. 2017 Jun 19;17:436. doi: 10.1186/s12879-017-2544-0 (PMC5477133; doi:10.1186/s12879-017-2544-0)
Supplement: Additional file 1: Table S1. — Survivals according to scores of Mycobacterium avium complex lung disease prognostic index. Survivals (1-year, 3-year, and 5-year) according to the prognostic scores are shown. (DOCX 14 kb) [file 12879_2017_2544_MOESM1_ESM.docx]

| Table S1 | | | | |
| --- | --- | --- | --- | --- |
| Survivals according to scores of *Mycobacterium avium* complex lung disease prognostic index | | | | |
| Scores of prognostic socres | N | Overall survival (%) | | |
|  |  | 1-year | 3-year | 5-year |
| 0 | 56 | 100 | 100 | 100 |
| 1 | 91 | 98.9 | 97.7 | 96.1 |
| 2 | 93 | 94.5 | 88.9 | 84.4 |
| 3 | 61 | 85.5 | 79.5 | 65.0 |
| 4 | 41 | 79.5 | 55.6 | 39.4 |
| 5 | 21 | 45.0 | 32.8 | 16.4 |
| 6 | 3 | 66.7 | NE | NE |
| 7 | 2 | NE | NE | NE |
| MPI, *Mycobacterium avium* complex lung disease prognostic index ; NE, not evaluated | | | | |
